# Current and Future Landscape of Hepatocellular Carcinoma Treatment

**DOI:** 10.32604/or.2026.076937

**Published:** 2026-05-21

**Authors:** Shadi Zerehpoosh, Yasuhito Tanaka, Said A. Al-Busafi, Gulnara Aghayeva, Samir Rouabhia, Qiuwei Pan, Mohammed Eslam

**Affiliations:** 1 Storr Liver Centre, Westmead Institute for Medical Research, Westmead Hospital and University of Sydney, Sydney, NSW, Australia; 2 Department of Gastroenterology and Hepatology, Faculty of Life Sciences, Graduate School of Medical Sciences, Kumamoto University, 1-1-1, Honjo, Chuo-Ku, Kumamoto, Japan; 3 Department of Medicine, College of Medicine and Health Sciences, Sultan Qaboos University, Muscat, Oman; 4 Medical Research Center, Sultan Qaboos University, Muscat, Oman; 5 Liver Diseases Department, Baku Health Center, Baku, Azerbaijan; 6 Department of Internal Medicine, University Hospital Center Touhami Benflis, Batna, Algeria; 7 Department of Gastroenterology and Hepatology, Erasmus MC-University Medical Center, Rotterdam, The Netherlands

**Keywords:** Hepatocellular carcinoma (HCC), immunotherapy, targeted therapy, combination therapy, systemic therapy

## Abstract

Hepatocellular carcinoma (HCC) represents a critical global health challenge, standing as a leading cause of cancer mortality with a significant and projected increasing incidence worldwide. A primary hurdle in HCC management is late diagnosis, often attributable to the absence of early symptoms. Despite considerable advancements in therapeutic strategies over the past decade, including immune checkpoint inhibitors and targeted therapies, mortality rates remain high, underscoring the urgent need for more effective novel approaches. The inherent molecular complexity and heterogeneity of HCC, where only a minority of tumors possess readily targetable drivers, contribute to treatment resistance and recurrence, even after curative interventions. Addressing these challenges, combination therapies, integrating immunotherapy with targeted agents, are emerging as a promising strategy to enhance efficacy and overcome resistance. This review comprehensively explores the current landscape of HCC treatment and the advancements in systemic therapies, including molecularly targeted agents and immunotherapies, and highlights ongoing challenges and future directions in this rapidly evolving field.

## 1 Introduction

Hepatocellular carcinoma (HCC) continues to be a formidable global health challenge, ranking as the sixth most common cancer [1]. With approximately 866,000 new cases and 759,000 deaths recorded in 2022, HCC accounts for the majority of primary liver cancers and remains the third leading cause of cancer-related mortality worldwide [1]. This malignancy is characterized by a markedly high fatality rate compared with most other cancers. Between 2010 and 2021, global incident cases and deaths increased by 26% and 25%, respectively, exceeding 866,000 new cases and 759,000 deaths in 2022, with projections estimating 1.4 million new cases annually by 2040 [2,3].

While viral hepatitis predominates, metabolic dysfunction-associated fatty liver disease (MAFLD) shows rising age-standardized incidence and mortality, increasing its global/regional HCC burden [4]. A major obstacle in HCC management is its frequent late-stage diagnosis, as patients often present with advanced, incurable tumors due to the absence of clear symptoms in the early stages [5,6,7].

Despite considerable advancements in therapeutic strategies over the past decade, including immune checkpoint inhibitors and targeted therapies that dramatically changed the management landscape of HCC, its mortality rates remain stubbornly high. This underscores an urgent need for more effective and durable therapeutic approaches to improve patient outcomes in advanced stages of the disease [8].

While genomic studies have elucidated many molecular alterations underlying HCC, a significant proportion of identified mutations are not readily targetable, with only approximately 28% of patients harboring at least one damaging alteration potentially targetable by an FDA-approved drug [9]. This highlights the critical need for a more comprehensive understanding of HCC’s molecular heterogeneity to develop personalized treatment strategies [10]. Given the limitations of single-agent approaches and the complex interplay of biological pathways in HCC, combination therapies integrating immunotherapy with targeted agents are gaining prominence as a promising avenue to enhance therapeutic efficacy and overcome resistance mechanisms [11,12].

The progression of HCC is intricately linked to underlying liver diseases, with chronic inflammation and fibrosis serving as critical precursors to malignant transformation. HCC is driven primarily by chronic viral hepatitis B and C infections, alongside growing contributions from MAFLD and alcoholic liver disease (ALD) [13,14,15,16]. Furthermore, metabolic abnormalities significantly influence the risk of HCC development in patients with hepatitis C [17]. HCC accounts for over 90% of all primary liver cancers, with a disproportionately higher incidence observed in men compared to women [18,19]. The regional variations in the primary etiological factors contributing to HCC are further detailed in Table 1.

**Table 1:** Regional variations in the primary etiological factors contributing to hepatocellular carcinoma (HCC), demonstrating how the burden of disease etiology shifts geographically.

Region	Predominant Etiological Factors and Their Contribution	Source
**Global**	HBV (~40%), HCV (~29%), ALD (~19%), MAFLD (~7%)	[20]
**Eastern Asia**	HBV (up to 62% of liver cancer-related deaths)	[20]
**Japan**	HCV (>70% of HCCs)	[21]
**North America**	HCV (~38%), ALD (~29%), HBV (~14%), MAFLD (~10%)	[22]
**Western Europe**	HCV (37% of HCCs overall); ALD and MAFLD increasing	[5]
**Central & Eastern Europe**	ALD (nearly 50% of HCCs)	[5]
**MENA**	HBV (~ 24%), HCV (~45%), ALD (~11%), MAFLD (~11%)	[23]

Abbreviations: HBV, hepatitis B virus; HCV, hepatitis C virus; ALD, alcohol-associated liver disease; MAFLD, metabolic dysfunction–associated fatty liver disease; HCC, hepatocellular carcinoma; MENA, Middle East and North Africa.

Even with curative interventions, the recurrence rate of HCC within five years can be as high as 80% [24]. Consequently, advanced HCC often necessitates systemic therapeutic approaches, which have evolved significantly from single-agent regimens to complex combination strategies [25,26]. Despite these advancements, drug resistance remains a significant hurdle in targeted therapy, often leading to treatment failure due to both primary and acquired resistance mechanisms [27]. This necessitates the ongoing development of novel agents and combination strategies that can circumvent these resistance pathways and provide more durable responses for patients with advanced HCC [28].

This review aims to synthesize the current landscape of HCC treatment, focusing on recent advancements in systemic therapies, including molecularly targeted agents and immunotherapies, and to explore future directions in this rapidly evolving field.

## 2 Search Strategy

To provide a comprehensive and up-to-date overview, we conducted a literature search in September 2025 using the PubMed (https://pubmed.ncbi.nlm.nih.gov) and EMBASE (https://www.embase.com) databases, focusing on publications in English. Our search strategy employed key terms related to core concepts in hepatocellular carcinoma, including Hepatocellular carcinoma, immunotherapy, targeted therapies, combination therapies, and systemic therapy.

The selection process prioritized original research articles, review articles, and clinical trial reports that provided insights into treatment strategies, emerging immunotherapeutic approaches, and advances in targeted therapies. Where high-quality human studies were limited, non-randomized studies and preclinical research were also considered, while acknowledging their inherent limitations. This approach was designed to provide a thorough narrative overview without applying a formal systematic review methodology.

## 3 Diagnosis and Staging of HCC

**Figure 1:**
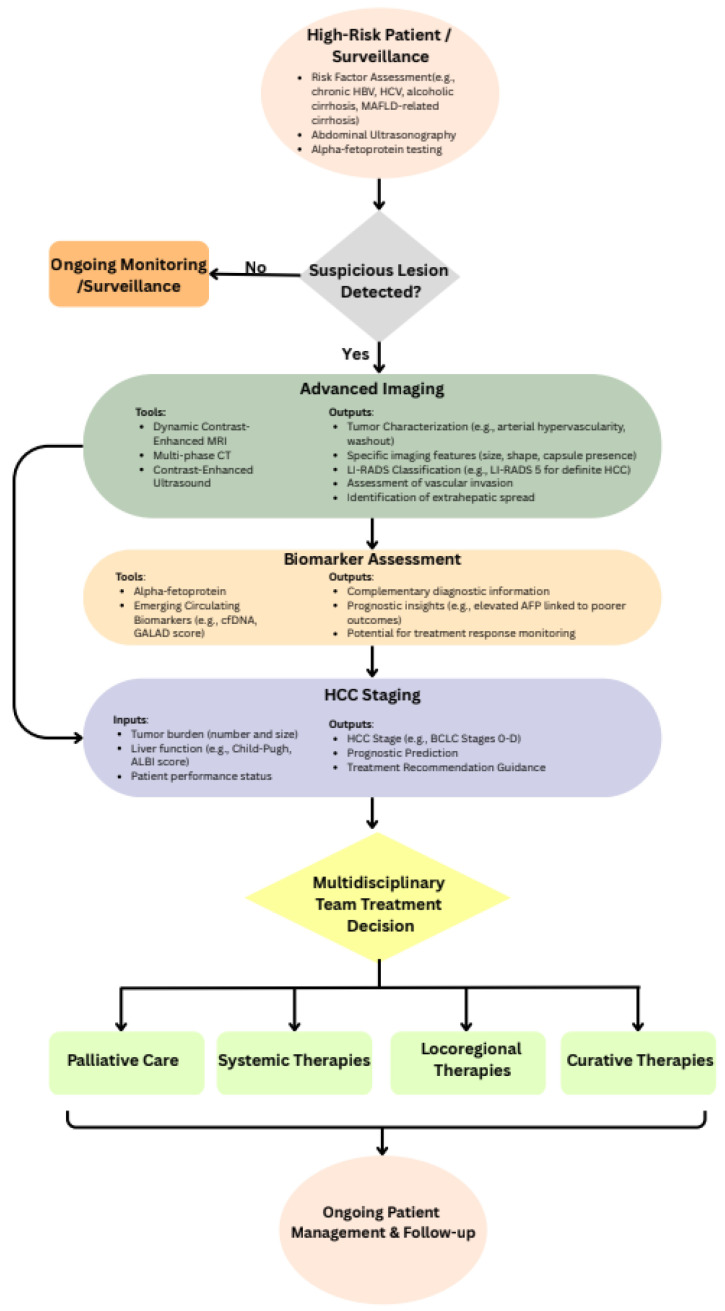
Proposed diagnostic and clinical decision-making pathway for Hepatocellular Carcinoma (HCC). This schematic outlines the recommended workflow for the detection, evaluation, and management of patients at risk of or diagnosed with HCC. The pathway integrates risk stratification, surveillance strategies, diagnostic imaging, biomarker assessment, and treatment decision points, from initial clinical suspicion through therapeutic allocation and follow-up [29,30].

Given HCC’s multifactorial etiology, early and accurate diagnosis, coupled with appropriate staging, is paramount for guiding therapeutic decisions and improving patient outcomes. The diagnostic and management workflow for HCC is summarized in Fig. 1.

### 3.1 Imaging Modalities

Advanced imaging techniques, including multiphasic computed tomography (CT) and magnetic resonance imaging (MRI) employing liver-specific contrast agents, are indispensable for the non-invasive detection, characterization, and staging of HCC [31]. These modalities allow for precise tumor localization, assessment of vascular invasion, and identification of extrahepatic spread, all crucial elements for clinical decision-making [32,33]. The application of contrast-enhanced ultrasound also provides real-time vascular dynamics, which can be particularly useful in characterizing smaller lesions and guiding biopsies [34].

### 3.2 Biomarkers and Molecular Diagnostics

While imaging remains central, the integration of established clinical serological biomarkers, notably alpha-fetoprotein (AFP), alongside regional standards like protein induced by vitamin K absence/antagonist-II (PIVKA-II) and the L3 fraction of AFP (AFP-L3%) (widely used in East Asia for HCC diagnosis, surveillance, and recurrence monitoring) [35,36,37], and multi-marker panels such as GALAD score (combining Gender, Age, AFP-L3%, AFP, and Des-gamma-carboxy prothrombin (DCP)), offers complementary insights into HCC presence, prognosis, and therapeutic response [38]. However, the sensitivity and specificity of AFP alone are limited, especially in early-stage HCC, with AFP serving as the primary evidence-based tool for guiding select treatment decisions globally, such as ramucirumab eligibility in advanced cases [39,40], necessitating the exploration of more accurate and multiplexed biomarker panels and approaches.

Recent research is exploring the identification of new circulating biomarkers, such as microRNAs, circulating tumor DNA, and specific changes in circulating metabolites, which show promise as predictors for patients with HCC. One candidate biomarker, aldo-keto reductase family 1 member B10 (AKR1B10), has been noted for its higher diagnostic accuracy compared to AFP alone; however, it is still mainly for investigational purposes and has not yet been incorporated into clinical practice [41]. Other biomarkers, such as des-γ-carboxy prothrombin and glypican-3 (GPC3), have also been studied for their potential in early diagnosis, but the results have been inconsistent. As a result, these biomarkers are not currently recommended for routine clinical use or for guiding treatment decisions [42,43].

Furthermore, advanced sequential algorithms, incorporating scores like ADAPT (combining Age, Diabetes, N-terminal propeptide of type III collagen [PRO-C3], and Platelet count) and liver stiffness measurements, are being developed for non-invasive staging of MAFLD, aiding in the identification of patients at risk for HCC and informing treatment stratification [44].

### 3.3 Staging Systems

Currently, the Barcelona Clinic Liver Cancer (BCLC) staging system is widely adopted for classifying HCC, as it integrates tumor burden, liver function, and patient performance status to guide treatment selection and predict prognosis [45,46]. This comprehensive system facilitates a stratified approach to therapy, enabling clinicians to tailor interventions from curative resection to palliative care based on individual patient profiles. However, despite its widespread use, the BCLC staging system, like other available staging systems, is not universally accepted due to variations in population cohorts and diverse HCC etiologies across different geographical regions [47]. To further illustrate the integrated approach of the BCLC staging system in guiding therapeutic decisions, Table 2 summarizes the recommended treatment options based on BCLC stage, liver function, and performance status [48].

**Table 2:** Integrated treatment algorithm for HCC by Barcelona Clinic Liver Cancer (BCLC) stage.

BCLC Stage	Tumor Characteristics	Liver Function	Performance Status	Recommended Treatment Options
**0**	Single ≤2 cm	Preserved	0	Primary: Thermal Ablation Alternative: Surgical Resection
**A**	Single or ≤3 nodules each ≤3 cm	Preserved	0	Single: Resection Multinodular: Liver Transplantation or Ablation
**B**	Multifocal (>3 nodules, or up to 3 nodules, with at least 1 >3 cm)	Preserved	0	TACE, Transplantation, Systemic Therapy
**C**	Vascular invasion and/or extrahepatic spread	Preserved	1–2	Systemic Therapy
**D**	Any	End Stage	3–4	Palliative Care

Abbreviations: BCLC, Barcelona Clinic Liver Cancer; TACE, transarterial chemoembolization.

## 4 Pathogenetic Mechanisms and Contributing Factors

### 4.1 Genetic and Epigenetic Alterations

The pathogenesis of HCC is complex and multifactorial, characterized by the aberrant activation of several molecular signaling pathways, abnormal hepatocellular differentiation, increased angiogenesis, and impaired immune function [49,50]. Significant genetic and epigenetic alterations are critical to the development and progression of HCC [51,52]. These alterations encompass chromosomal amplifications and deletions, as well as the epigenetic inactivation of tumor suppressor genes through mechanisms such as DNA hypermethylation and histone modifications [53,54]. Genetic variants, such as those in the major histocompatibility complex (MHC) class I polypeptide–related sequence A (MICA) gene, have also been implicated in driving liver fibrosis progression in chronic hepatitis C through TGF-β-dependent mechanisms [55].

### 4.2 Dysregulated Signaling Pathways

Key signaling pathways frequently dysregulated in HCC include the Wnt/β-catenin pathway, which is crucial for normal liver development and regeneration. However, its aberrant activation drives uncontrolled cell proliferation and survival, thereby promoting HCC [56]. Mutations in the Catenin Beta 1 gene, are observed in a significant proportion of HCC cases, leading to stabilized β-catenin and its nuclear translocation [57,58]. This activation significantly enhances the proliferation and invasion of HCC cells and contributes to therapeutic resistance [57].

Dysregulation of the PI3K/Akt/mTOR signaling pathway is a common feature in HCC, playing a central role in tumor cell proliferation, growth, survival, and angiogenesis [59]. Aberrant activation of this pathway is often correlated with a higher tumor grade and worse prognosis [60], and it can also confer resistance to therapies such as sorafenib [10]. Consequently, targeting this pathway, particularly mTOR, offers a strategy to suppress tumor cell growth [61,62,63].

Aberrantly activated Janus kinase–signal transducer and activator of transcription (JAK–STAT) signaling leads to the malfunction of downstream target genes, which are critical for controlling cell survival, division, angiogenesis, and metastasis in HCC [64]. Elevated activation of STAT proteins in tumors compared to adjacent liver tissues is strongly associated with poor prognosis [65]. This pathway further contributes to the maintenance of cancer stem cells and fosters an immunosuppressive microenvironment [66].

The Mitogen-Activated Protein Kinase (MAPK) pathway, particularly the Extracellular Signal–Regulated Kinase (ERK) signaling cascade, is deeply implicated in HCC pathogenesis, promoting cell proliferation and driving HCC development [67]. Activating mutations in RAS and RAF, along with the inactivation or repression of endogenous RAS regulators, are frequently observed in many cancers, including HCC [68].

**Figure 2:**
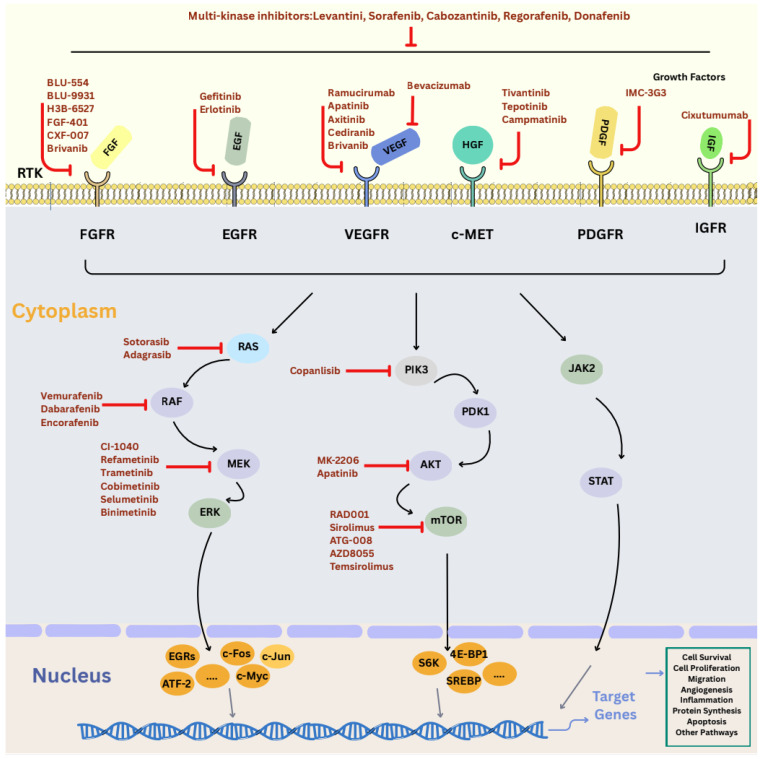
Major signaling pathways and molecular targets involved in HCC. This diagram summarizes the key oncogenic signaling cascades implicated in HCC development and progression, including receptor tyrosine kinase (RTK)-mediated pathways, downstream effectors, and representative molecular targets. Pathways commonly dysregulated in HCC are depicted along with therapeutic nodes [50]. Abbreviations: Fibroblast Growth Factor (FGF/FGFR), Epidermal Growth Factor (EGF/EGFR), Vascular Endothelial Growth Factor (VEGF/VEGFR), Hepatocyte Growth Factor (HGF), Platelet-Derived Growth Factor (PDGF/PDGFR), Insulin-Like Growth Factor (IGF/IGFR), c-MET, RAS, RAF, MAPK/ERK Kinase (MEK), Extracellular Signal–Regulated Kinase (ERK), Phosphoinositide 3-Kinase (PI3K), 3-Phosphoinositide–Dependent Protein Kinase 1 (PDK1), Protein Kinase B (AKT), Mechanistic Target of Rapamycin (mTOR), Janus Kinase 2 (JAK2), Signal Transducer and Activator of Transcription (STAT), Early Growth Response proteins (EGRs), Cellular Fos (c-Fos), Cellular Jun (c-JUN), Activating Transcription Factor 2 (ATF-2), Cellular Myelocytomatosis (c-Myc), Eukaryotic Translation Initiation Factor 4E-Binding Protein 1 (4E-BP1), Ribosomal Protein S6 Kinase (S6K), Sterol Regulatory Element-Binding Protein (SREBP).

The Programmed Cell Death Protein 1–Programmed Death-Ligand 1 (PD1-PDL1) axis is another critical pathway involved in HCC progression, primarily by enabling immune evasion within the tumor microenvironment. PD 1 and its ligand, PDL 1, regulate T-cell activity, and their aberrant expression in HCC allows cancer cells to evade immune surveillance, fostering tumor growth and metastasis [69]. The therapeutic success of checkpoint inhibitors targeting this pathway underscores its significant role in HCC pathogenesis. Key oncogenic signaling pathways and molecular targets relevant to HCC treatment are depicted in Fig. 2.

### 4.3 Additional Contributing Mechanisms

Beyond these pathways, other significant alterations contributing to HCC progression involve telomere maintenance, cell cycle control, chromatin modification, and oxidative stress [69]. Additionally, epigenetic mechanisms such as the overexpression of IGF2 and silencing of CDKN2A are recognized contributors to HCC tumorigenesis [70].

## 5 Current Treatment Landscape of HCC

The current treatment landscape for HCC is complex and rapidly evolving, encompassing a wide array of modalities tailored to the stage of disease, liver function, and patient characteristics.

### 5.1 Curative and Early-Stage Treatment Modalities

For patients presenting with early-stage HCC, potentially curative interventions such as surgical resection, liver transplantation, and locoregional ablative therapies offer the best prospects for long-term survival.

#### 5.1.1 Surgical Resection

Surgical resection is considered a potentially curative option for early-stage HCC, particularly for patients with a solitary mass, adequate liver reserve, and liver function that is either non-cirrhotic or classified as Child-Pugh A without clinically significant portal hypertension [70]. While it offers promising overall survival rates of 70–80%, the recurrence rate can be as high as 70% [71].

The decision for resection depends on an appropriate tumor location that allows for a sufficient liver remnant after surgery [70]. Minimally invasive approaches, including laparoscopic and robotic techniques, have become standard, leading to more robust liver remnants, reduced surgical risks, fewer complications, and faster recovery.

Preoperative portal vein embolization can also be used to induce hypertrophy of the future liver remnant, expanding the eligibility for surgery. Despite its promise, strict eligibility requirements limit curative-intent surgery to roughly one-third of evaluated patients [71,72].

#### 5.1.2 Liver Transplantation

Liver transplantation is another curative treatment modality for HCC, especially for selected patients with underlying cirrhosis, as it not only cures HCC but also eliminates the underlying cirrhotic liver, thereby reducing the risk of recurrence [70,73]. Patients within the Milan criteria (solitary lesion less than 5 cm or up to three lesions, none greater than 3 cm, without vascular invasion or distant metastases) are considered ideal candidates [74].

Liver transplantation in these patients boasts impressive 5-year overall survival rates often reaching 70% to 85% and recurrence-free survival rates of 92%. While organ availability and strict eligibility criteria limit its widespread application [75,76,77], expanded criteria, such as the University of California, San Francisco (UCSF) criteria or “up-to-seven”, have been adopted to broaden the pool of eligible patients [75,78]. Locoregional therapies can also be used for downstaging to meet transplant criteria, a practice recognized by United Network for Organ Sharing (UNOS) [79,80], and for eligible patients, the five-year overall survival with LT significantly surpasses that of other locoregional or systemic therapies (77.5% vs. 31.2%) [73]. The Model for End-Stage Liver Disease (MELD) assesses the severity of chronic liver disease to prioritize patients for liver transplantation. MELD exception points are typically awarded for HCC lesions at least 2 cm in size, with current UNOS policy requiring an AFP level of <1000 ng/mL for eligibility, reduced to <500 ng/mL after locoregional therapy for those with initial AFP ≥ 1000 ng/mL [74,81].

#### 5.1.3 Ablation

Ablation techniques, such as radiofrequency ablation (RFA) and microwave ablation, are crucial for managing early-stage HCC, especially for patients ineligible for surgery or when liver transplantation and resection are contraindicated. These methods are most effective for small tumors, ideally less than 3 cm, with effectiveness and survival inversely proportional to tumor dimensions [82,83].

Ablation destroys tumor cells with thermal energy (or freezing in cryoablation) and is employed not only for early-stage disease but also for intermediate-stage HCC, as well as for downstaging or serving as a bridge to liver transplantation.

Cryoablation offers unique advantages, including visualization of the ice ball, minimal procedural pain, and strong immunomodulatory effects that modulate the tumor immune microenvironment and induce systemic anti-tumor immune responses [84,85].

It offers survival comparable to resection for small tumors, with reduced complication rates and cost-effectiveness. Tumor location is critical; areas near major blood vessels or bile ducts (due to the “heat sink effect”) and dome lesions near the diaphragm (due to thermal injury risk) are generally not ideal [86]. Ablation can also be used for downstaging or as a bridge to transplantation. Despite consistently achieving complete response rates over 90% and a median overall survival of approximately 60 months, RFA is associated with 5-year recurrence rates of 43–70% for small tumors [87,88].

### 5.2 Other Locoregional Therapies

Other locoregional therapies, including transarterial chemoembolization (TACE), and stereotactic body radiation therapy (SBRT), are crucial for managing intermediate-stage HCC or bridging patients to transplantation [89]. These minimally invasive procedures directly target tumors, reducing systemic toxicity and preserving liver function.

TACE remains the standard of care for BCLC stage B disease. It delivers chemotherapy agents directly to the tumor via the hepatic artery, followed by embolization to induce ischemic necrosis. Although primarily palliative, TACE has been shown to improve overall survival and tumor response rates in appropriately selected patients [90,91].

SBRT delivers highly precise external beam radiation, offering an alternative for patients with unresectable tumors or those ineligible for other locoregional treatments. It has demonstrated favorable local control and tolerability, even in patients with compromised hepatic reserve [92].

Despite their efficacy, these therapies are generally palliative rather than curative, and their effectiveness can be limited by tumor size, multifocality, and vascular invasion [93]. Ongoing research continues to refine combination strategies, including sequential or concurrent use with systemic therapies, to enhance local control and survival outcomes in intermediate and advanced HCC.

### 5.3 Systemic Therapies

**Figure 3:**
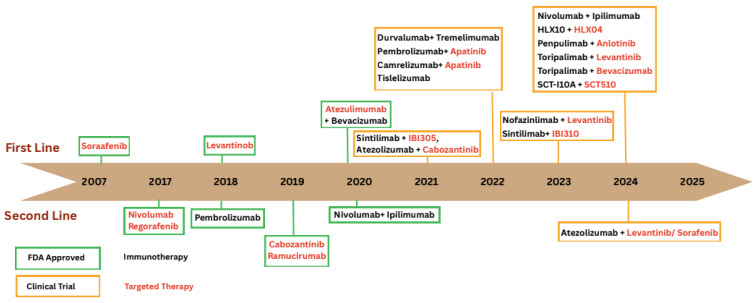
Timeline of approved and emerging systemic therapies for HCC. This figure illustrates the chronological development of systemic treatment options for HCC, distinguishing between first line (upper panel) and second line (lower panel) therapies introduced from 2007 to 2024. Food and Drug Administration (FDA)-approved therapies are displayed within green frames, while agents currently in Phase III clinical trials appear within orange frames. Targeted therapies are shown in red text and immunotherapies in black.

Systemic therapies have transformed the management of advanced HCC, providing significant survival benefits for patients with unresectable diseases, macrovascular invasion, or extrahepatic metastases. These treatments aim to target specific molecular pathways involved in tumor growth, angiogenesis, and immune evasion, though the development of resistance is a common challenge that necessitates sequential or combination approaches. The evolution of approved and investigational systemic therapies for HCC [94] is illustrated in Fig. 3.

#### 5.3.1 Sorafenib

Sorafenib was the first approved systemic therapy to demonstrate a survival benefit in advanced HCC in 2007 [95]. It is an orally active multi-kinase inhibitor that targets both tumor cell proliferation and angiogenesis [95,96]. Its mechanism involves blocking the Ras/Raf/MEK/ERK pathway and inhibiting angiogenesis by targeting full-term VEGF receptors and platelet-derived growth factor receptor β. Sorafenib also exhibits anti-proliferative, anti-angiogenic, and pro-apoptotic properties [97].

In the SHARP trial, sorafenib showed a median overall survival of 10.7 months compared to 7.9 months in the placebo group [95,97]. A similar survival benefit was observed in a parallel trial in the Asian-Pacific population [98].

Sorafenib is recommended as a standard first-line systemic therapy for patients with advanced HCC, BCLC-C stage, or those who have progressed after locoregional therapies, provided they have well-preserved liver function. Common adverse effects include diarrhea and hand-foot skin reaction, with the appearance of dermatologic reactions sometimes linked to better survival [95].

Sorafenib’s systemic exposure causes significant toxicities in approximately 50% of patients, frequently requiring dose reduction or discontinuation. Clinical trials reported 80% treatment-related adverse events (vs. 52% for placebo), with grade 3 diarrhea and hand-foot skin reactions notably frequent [95]. Real-world data indicate approximately 34% of patients experience grade 3/4 adverse effects, such as fatigue, diarrhea, hand-foot skin reaction, and hypertension. Also, serious adverse effects and higher mortality are more common in Child-Pugh B patients [99,100,101].

#### 5.3.2 Lenvatinib

Lenvatinib, a potent multi-kinase inhibitor, was introduced in 2018 as a crucial first-line therapy for unresectable HCC, following a decade where sorafenib was the sole option. Lenvatinib targets multiple receptors, including full term VEGFR1-3, FGFR1-4, PDGFR-α, RET, and KIT [102]. The REFLECT study, a phase III non-inferiority trial, evaluated lenvatinib against sorafenib as a first-line treatment for unresectable HCC. Lenvatinib demonstrated non-inferiority in overall survival, achieving a median overall survival of 13.6 months compared to 12.3 months for sorafenib [103].

Notably, lenvatinib also demonstrated notable improvements in objective response rates, achieving 24.1% compared to 9.2% for sorafenib [46]. A subgroup analysis of the REFLECT trial further indicated an even better median overall survival of 22.4 months for patients who initially responded to lenvatinib. As a result, lenvatinib is now recognized as an approved first-line systemic therapy for unresectable HCC. In patients with MAFLD-related HCC, lenvatinib showed numerically better median progression-free survival and overall survival compared to patients without MAFLD, with no significant difference in tumor response or adverse effects occurrence [103,104].

Despite its efficacy, lenvatinib is associated with a higher incidence of grade ≥ 3 treatment-related adverse events compared to sorafenib. The most common adverse effects of any grade include hypertension, diarrhea, decreased appetite, and decreased bodyweight, while grade 3/4 adverse effects frequently involve hand-foot skin toxicity, hypertension, and diarrhea. Treatment discontinuation due to adverse effects occurred in 9% of patients [105].

#### 5.3.3 Regorafenib

Regorafenib is a tyrosine kinase inhibitor with anti-angiogenic properties, similar to sorafenib, and is approved for the treatment of HCC patients who have been previously treated with sorafenib [8]. Its mechanism involves inhibiting VEGF receptor, PDGF receptor, FGF receptor, RAF, RET, and KIT. It also blocks cell growth and invasion, targets the MAPK pathway, and activates apoptotic pathways [106].

The RESORCE trial, a phase III clinical trial, demonstrated that oral regorafenib administration significantly improved overall survival in a second line setting for patients who had progressed on sorafenib. The median overall survival for the regorafenib arm was 10.6 months, compared to 7.8 months for the control group. The median time to progression was 3.2 months with regorafenib versus 1.5 months with placebo [107].

However, regorafenib treatment leads to a substantial incidence of adverse events, with grade 3 or 4 treatment-related adverse effects occurring in 50% of patients. The most common adverse events associated with regorafenib are hand-foot skin reaction, diarrhea, fatigue, hypertension, and anorexia [108].

#### 5.3.4 Cabozantinib

Cabozantinib is a multi-kinase inhibitor that targets VEGFR1-3, hepatocyte growth factor/c-MET, RET, KIT, FLT-3, TIE2, and AXL [109]. It is unique among some multikinase inhibitors in its ability to block c-Met, which has been linked to resistance to sorafenib. Cabozantinib has shown anti-tumor activity by inhibiting tumor growth, angiogenesis, invasion, and migration [110,111]. Based on the randomized phase III CELESTIAL trial, cabozantinib is approved for second-line treatment of HCC after progression on sorafenib in patients with unresectable HCC. In this trial, cabozantinib achieved a median overall survival of 10.2 months compared to 8.0 months in the placebo group, with a progression-free survival of 5.2 months versus 1.9 months [111,112].

Cabozantinib therapy has high rates of adverse events. The CELESTIAL trial reported Grade 3 or 4 adverse effects in 68% of cabozantinib patients (vs. 36% placebo), leading to dose reductions for most and discontinuation for 16% [112,113]. Common adverse effects were diarrhea, decreased appetite, palmar-plantar erythrodysesthesia, fatigue, and hypertension.

#### 5.3.5 Ramucirumab

Ramucirumab is a human monoclonal antibody that specifically binds to the VEGF receptor 2. By blocking the binding of ligands like VEGF-A, VEGF-C, and VEGF-D to VEGFR2, ramucirumab prevents tumor angiogenesis [114,115]. The REACH-2 trial, a double-blind phase III study, showed that ramucirumab extends survival in a subgroup of HCC patients with elevated baseline AFP levels who had progressed on sorafenib. For these patients, ramucirumab treatment resulted in a median overall survival of 8.5 months, exceeding the 7.3 months observed in placebo arm, and increased progression-free survival (PFS) to 2.8 months from 1.6 months. Consequently, ramucirumab was approved for second-line treatment in advanced HCC patients with AFP ≥ 400 ng/mL [116].

Ramucirumab’s safety profile includes adverse events, with hypertension being the most common grade 3 or 4 adverse event, and hyponatremia occurring in 5.6% of patients. A real-world study reported a discontinuation rate due to adverse effects of 29.4%, and three patients in the REACH-2 trial died from treatment-emergent adverse effects related to the study treatment [117].

#### 5.3.6 Immunotherapies

Immunotherapies, particularly immune checkpoint inhibitors, have demonstrated significant potential as a treatment approach for HCC by strategically engaging the immune system. These treatments target immune checkpoint proteins such as full term PD1, CTLA4, TIGIT, and LAG3 on T cells. By inhibiting these checkpoints, ICIs facilitate the activation of the patient’s immune response, leading to the destruction of malignant cells [118].

Although monotherapy with PD1/PDL1 inhibitors did not demonstrate superior overall survival compared to sorafenib in certain first-line investigations, yet encouraging response rates, durability, and safety profiles were observed [119]. For instance, in the Checkmate 459 study, while nivolumab did not demonstrate a statistically significant difference in overall survival compared to sorafenib in the first-line setting, it exhibited superior response rates and a more favorable safety profile, characterized by a reduced incidence of grade 3–4 adverse events [120]. Combination immunotherapy has shown promise. For eligible patients with advanced HCC (Child-Pugh A liver function, ECOG Performance Status 0–1), atezolizumab in combination with bevacizumab and durvalumab plus tremelimumab have become the preferred first-line systemic therapy options, demonstrating superior efficacy compared to previous standards like sorafenib [121]. Similarly, the HIMALAYA trial demonstrated that the combination of durvalumab and tremelimumab significantly improved overall survival when compared to sorafenib [122].

Dual immune checkpoint blockade and combinations of ICIs with targeted agents are currently under investigation in large, randomized Phase 3 trials, aiming to further expand therapeutic options. The presence of tumor-infiltrating lymphocytes in HCC tumors suggests the importance of immune responses in treatment, although the chronic inflammatory environment and tolerogenic tumor microenvironment of HCC can pose challenges [123].

Despite their promise, immune checkpoint inhibitors are associated with immune-related adverse events (irAEs), which are generally manageable but can occasionally result in fatal outcomes. The incidence of irAEs is relatively high in liver cancer patients [124]. A particular challenge in HCC patients is that symptoms of underlying liver diseases, such as cirrhosis from viral hepatitis or MAFLD, can overlap with irAEs, potentially leading to delayed recognition and inappropriate management [125].

Furthermore, response to ICIs has been reported to be impaired in patients with underlying MAFLD [126], reflecting regional etiological heterogeneity where viral HCC (predominant in Eastern Asia/MENA) responds better than non-viral HCC (rising in the West) [127]. Studies indicate that patients with non-viral etiology HCC, which includes MAFLD-HCC, may not benefit as much from ICI therapy as those with HBV- or HCV-related HCC, with non-viral etiology being associated with shorter survival in patients receiving anti-PD-1 therapy [128]. This suggests a potentially lower efficiency of immunotherapies in MAFLD-related HCC.

The differences in treatment responses necessitate etiology-stratified trial designs and region-tailored treatment selection to optimize immunotherapy efficacy. Following progression on first-line ICI-based regimens, such as atezolizumab–bevacizumab or durvalumab–tremelimumab, treatment sequencing remains guided largely by expert consensus and real-world evidence rather than prospective trials. Current guidelines recommend TKIs, including regorafenib, cabozantinib, or ramucirumab (for AFP ≥ 400 ng/mL), with agent selection influenced by prior therapy, liver function, toxicity profiles, and tumor characteristics. However, the optimal sequencing strategy following ICI failure remains undefined, underscoring the need for dedicated clinical trials [129,130].

## 6 Emerging Therapeutic Strategies and Future Directions

The landscape of HCC treatment is continuously evolving, driven by the imperative to overcome the limitations of current therapies, address drug resistance, and improve patient outcomes. Despite significant advancements in systemic treatments, their efficacy is not universal, and improvements in patient outcomes have often been modest and incremental, highlighting an unmet medical need for novel therapies [131]. An overview of emerging therapeutic strategies and novel molecular targets in HCC is summarized in Table 3.

Drug resistance, both primary and acquired, remains a major reason for treatment failure and can significantly limit therapeutic options, leading to poor prognoses for many HCC patients [132]. This necessitates the development of next-generation approaches, innovative combinations, and refined strategies to enhance anti-tumor immunity within the complex tumor microenvironment.

**Table 3:** Emerging therapeutic strategies and novel targets in HCC.

Type of Therapy	Novel Agent/Target	Mechanism/Targeted Pathway	Source
**Immune Checkpoint Inhibitors**	TIGIT inhibitors	Immune checkpoint inhibition	[133]
	LAG-3 inhibitors	Immune checkpoint inhibition	[133]
	TIM-3 inhibitors	Immune checkpoint inhibition	[133]
	ADG126	Masked anti-CTLA4 monoclonal antibody for selective depletion of regulatory T cells	[134]
	VISTA inhibitors	Modulating immune cell activity	[134]
	B7-H3 inhibitors	Modulating immune cell activity (B7-H3)	[134]
	B7-H4 inhibitors	Modulating immune cell activity (B7-H4)	[134]
	Tiragolumab	Anti-TIGIT antibody, enhances atezolizumab/bevacizumab efficacy	[134]
**Molecular Targeted Therapies**	Ferroptosis inducers	Induces regulated cell death via iron-dependent lipid peroxidation, blocking GPX4	[28]
	Glypican-3 targeted agents	Targets GPC3, a membrane-associated proteoglycan specifically upregulated in HCC	[36]
	Fibroblast Growth Factor Receptor pathway inhibitors	Targets aberrant FGF/FGFR signaling involved in HCC carcinogenesis, proliferation, angiogenesis	[135]
	Wnt/β-catenin pathway modulators	Targets key signaling pathway in HCC pathogenesis	[135]
	PI3K/Akt/mTOR pathway inhibitors	Targets key signaling pathway in HCC pathogenesis	[135]
	c-MET inhibitors	Targets c-MET pathway, involved in HCC pathogenesis	[134]
	CSF-1/CSF-1R axis modulators	Targets colony-stimulating factor-1/receptor, involved in macrophage activity and HCC progression	[134]
	Notch signaling pathway modulators	Targets Notch pathway, critical for angiogenesis and cell regulation in HCC	[134]
	Post-translational modification inhibitors	Targets various PTMs affecting key proteins in HCC development and resistance	[134]
**Advanced Combination Therapies**	ICI + TKI Combinations	Combines immune checkpoint inhibition with tyrosine kinase inhibition to enhance anti-tumor effects and overcome resistance	[25]
	Bispecific Antibodies	Targets two different pathways simultaneously (e.g., immune checkpoints and angiogenesis)	[136]
	Triplet Systemic Therapy (ICI + anti-VEGF + second-generation ICI)	Multi-pronged approach to address complex HCC nature and resistance	[133]
	Atezolizumab + Bevacizumab + Tiragolumab	Standard of care (Atezo + Bev) with an anti-TIGIT antibody	[133]
	Anti-PD-1 mAb + anti-LAG-3 mAb + anti-VEGF-A mAb	Triple combinations targeting multiple immune and angiogenic pathways	[133]
	Tremelimumab + Durvalumab + Cabozantinib	Combines dual ICI with a multi-kinase inhibitor	[137]
	Sintilimab + IBI310 + Bevacizumab	Combines dual ICI (PD-1 + CTLA-4) with an anti-VEGF antibody	[137]
	Coformulated Pembrolizumab/Quavonlimab + Lenvatinib	Combines coformulated dual ICI (PD-1 + CTLA-4) with a multi-kinase inhibitor	[137]
	SRF388 + Atezolizumab + Bevacizumab	Combines anti-IL-27 mAb with standard of care (Atezo + Bev)	[137]
**Cell-Based & Other Novel Therapies**	CAR-T cell therapies	Cell-based immunotherapy targeting HCC-specific antigens	[138]
	Oncolytic viruses	Novel therapeutic avenue that selectively replicates in and kills cancer cells	[139]

Abbreviations: TIGIT, T cell immunoreceptor with Ig and ITIM domains; LAG-3, lymphocyte activation gene-3; TIM-3, T cell immunoglobulin and mucin domain-3; CTLA-4, cytotoxic T-lymphocyte–associated protein 4; VISTA, V-domain Ig suppressor of T cell activation; GPX4, glutathione peroxidase 4; GPC3, glypican-3; FGF, fibroblast growth factor; FGFR, fibroblast growth factor receptor; PI3K, phosphoinositide 3-kinase; Akt, protein kinase B; mTOR, mechanistic target of rapamycin; c-MET, mesenchymal–epithelial transition factor receptor; CSF-1, colony-stimulating factor-1; CSF-1R, colony-stimulating factor-1 receptor; PTM, post-translational modification; ICI, immune checkpoint inhibitor; TKI, tyrosine kinase inhibitor; VEGF, vascular endothelial growth factor; PD-1, programmed cell death protein 1; IL-27, interleukin-27; CAR-T, chimeric antigen receptor T-cell; mAb, monoclonal antibody.

### 6.1 Strategies for Overcoming Resistance and Enhancing Efficacy

Despite progress, less than one-third of patients with HCC achieve an objective response to current immune checkpoint inhibitor-based treatments, highlighting the need for therapeutic optimization [140]. Resistance to both targeted therapies and immunotherapies is a complex, multifactorial challenge, encompassing both primary resistance (inherent non-responsiveness) and acquired resistance (development over time, often within six months for TKIs) [141,142].

This resistance stems from both tumor-extrinsic mechanisms, such as the immunosuppressive tumor microenvironment, impaired T-cell function, the liver’s inherent tolerogenic properties, and factors like M2 macrophages and the gut microbiome [131,140,142]. It also arises from tumor-intrinsic mechanisms, including tumor heterogeneity, genetic variations, the activation of alternative signaling pathways (e.g., EGFR, CDK6), and the influence of cancer stem cells and microRNAs [131].

Emerging strategies aim to tackle these challenges by modulating the tumor microenvironment, targeting novel pathways, and leveraging synergistic combination approaches. For instance, optimizing strategies to enhance the efficiency of immune checkpoint inhibitors in HCC includes improving antigen presentation, increasing T-cell recruitment and infiltration, and boosting the recognition of effector immune cells [143].

These approaches also extend to the exploration of novel agents that can overcome specific resistance mechanisms and the development of personalized cancer vaccines targeting neoantigens to further enhance immune activation. Further research is needed to validate proposed biomarkers and understand resistance mechanisms to immunotherapy in HCC, ultimately aiding in identifying patient responders and avoiding unnecessary side effects.

### 6.2 Novel Immune Checkpoint Targets

Beyond the established PD-1/PD-L1 and CTLA-4 pathways, research is actively exploring other immune checkpoints to further disarm the immunosuppressive tumor microenvironment and unleash stronger anti-tumor immune responses [131].

Recent findings in broader cancer contexts highlight ICI combination strategies (e.g., ICI + TKI, ICI + anti-VEGF) to enhance efficacy [133], resistance mechanisms like T-cell exhaustion and β-catenin signaling observed in other tumors [133,144], and biomarker concepts addressing PD-L1 limitations, TMB, and immune-excluded tumors [145]. These principles directly inform HCC strategies, where second-generation ICIs include inhibitors targeting TIGIT, LAG-3, and TIM-3, which are being explored, often in combination, to enhance anti-tumor immunity [146]. Notably, TIGIT overexpression, frequently driven by its ligand CD155, can suppress T-cell function and contribute significantly to immune escape within the tumor microenvironment [147].

ADG126, a masked anti-CTLA4 monoclonal antibody, is designed for selective depletion of regulatory T cells in target tissues with reduced off-target toxicity. It is currently being evaluated in a Phase 1b/2 study for first-line HCC, in combination with atezolizumab and bevacizumab [148]. Other emerging checkpoint targets, such as VISTA, B7-H3, and B7-H4, offer additional avenues for therapeutic intervention by modulating immune cell activity [149]. These novel targets hold promise for overcoming resistance mechanisms associated with first-generation ICIs and paving the way for more tailored and effective immunotherapeutic strategies in HCC.

### 6.3 Novel Molecular Targets and Pathways

The identification and targeting of specific molecular vulnerabilities in HCC continue to be critical areas of research, addressing aspects not fully managed by existing therapies. Emerging strategies involve modulating pathways such as the Fibroblast Growth Factor Receptor pathway, with inhibitors like Fisgatinib showing promise [150]. GPC3 is also gaining attention as a prominent tumor-associated antigen and therapeutic target, particularly given its association with resistance to PD-1 blockade [151].

Key signaling pathways integral to HCC pathogenesis, including the Wnt/β-catenin pathway, the c-MET pathway, and the PI3K/Akt/mTOR pathway, remain significant focuses for novel therapeutic interventions. Furthermore, ferroptosis is recognized as a new promising target for inducing regulated cell death [152], and various post-translational modifications are being explored for their roles in HCC development and treatment resistance [153].

Other avenues include targeting the CSF-1/CSF-1R axis to modulate macrophage activity [154], and the Notch signaling pathway, critical for angiogenesis and cell regulation in HCC [155]. Moreover, strategies that focus on overcoming intrinsic tumor factors contributing to immunosuppression, such as MDM2 blockage or EPHA2 deletion, are also under investigation to sensitize HCC cells to immunotherapy [156,157].

Additionally, ongoing research explores manipulating the gut microbiome, which has shown to influence the efficacy of immune checkpoint inhibitors by modulating bile acid composition. Given the rising incidence of hepatocellular carcinoma arising in the background of MAFLD, research into specific molecular vulnerabilities and therapeutic targets for these subtypes is becoming increasingly crucial. MAFLD-related HCC, often detected at late stages due to the lack of clear surveillance definitions beyond cirrhotic patients, presents unique challenges in treatment [158,159].

The pathogenesis of MAFLD-related HCC is driven by factors such as insulin resistance, autophagy, obesity, and gut microbiota imbalances, leading to chronic inflammation, altered lipid metabolism, and ultimately HCC development [160]. While cirrhosis is a significant risk factor, MAFLD-HCC can also develop in non-cirrhotic individuals [158].

In MAFLD-related HCC, the upregulation of pivotal molecular pathways, such as the MAPK and PI3K-Akt-mTOR pathways, plays a crucial role in promoting cellular survival and proliferation [161]. MAFLD-associated HCC frequently exhibits higher rates of ACVR2A mutations and is characterized by enriched bile and fatty acid signaling, oxidative stress, and inflammation, often falling into the Wnt/TGF-β proliferation subclass [162,163]. The unique pathophysiology of MAFLD-related HCC suggests that traditional immunotherapies may not be as effective in these patients, possibly due to aberrant T-cell activation [164]. This highlights a critical unmet need for developing specific biomarkers and tailored therapeutic options for this growing HCC subgroup.

### 6.4 Advanced Combination Therapies

The most promising future directions in HCC treatment involve combining different therapeutic modalities to enhance anti-tumor effects and overcome resistance, often by modulating the tumor microenvironment to increase susceptibility to other agents. A prime example is the success of TKI-immunotherapy combinations, such as atezolizumab with bevacizumab, which established a paradigm by simultaneously targeting angiogenesis and the immune system for superior clinical benefits [165]. This strategy is being widely investigated, with numerous ongoing clinical trials exploring new TKI-immunotherapy combinations, including lenvatinib with pembrolizumab [166] and cabozantinib with nivolumab, even in neoadjuvant settings [167].

Recent phase III results from the COSMIC-312 trial confirmed the superiority of PFS for cabozantinib combined with atezolizumab compared to sorafenib [168]. Additionally, case reports indicate that the combination of atezolizumab and bevacizumab can enable conversion surgery in patients with unresectable cases, with pathological complete responses reported after treatment [169,170]. Furthermore, integrating multimodal approaches with locoregional options, such as TACE, further supports the goal of curative intent in intermediate-stage disease [171].

Another innovative approach involves bispecific antibodies, which are designed to simultaneously target two different pathways. PD-1/VEGF bispecific antibodies have shown promising efficacy in clinical trials by blocking both PD-1 and VEGF pathways [172]. Similarly, PD-1/CTLA-4 bispecific antibodies are being evaluated, demonstrating favorable objective response rates when combined with lenvatinib [173]. Additionally, PD-1/LAG-3 bispecific antibodies have exhibited anti-tumor activity in HCC patients [174].

Looking ahead, the future of first-line HCC treatment for advanced metastatic disease appears to be moving towards triplet systemic therapy. This multi-pronged approach typically combines an immune checkpoint inhibitor, an anti-VEGF agent, and a second-generation ICI, aiming to address the complex nature of HCC and its resistance mechanisms from multiple angles simultaneously.

### 6.5 Expanding Role in Early-Stage and Perioperative Settings

While much of the research on immune checkpoint inhibitor-based therapies has focused on advanced HCC, there is growing interest in extending their use to earlier disease stages, including neoadjuvant and adjuvant settings [175]. The mechanistic rationale for employing ICIs in these contexts is predicated on the ability to engage the immune system when the tumor burden might be more manageable, thereby potentially overcoming the challenges posed by large primary tumors and micro metastases. Moving ICIs into the perioperative setting for resectable disease represents an active and crucial area of investigation, especially given the significant unmet need for effective adjuvant or neoadjuvant therapies for HCC.

Early studies investigating neoadjuvant ICIs have shown promising results, with major pathological responses observed in a significant proportion of patients [167]. This approach aims to reduce tumor size and improve resectability before surgery, or to eliminate residual disease after resection, ultimately enhancing long-term outcomes. Furthermore, locoregional therapies, such as radiation therapy, may play a role in priming the tumor microenvironment, making it more susceptible to immunotherapy and potentially boosting treatment response. The successful application of neoadjuvant and adjuvant therapies in other cancers, like melanoma and non-small cell lung cancer, sets a precedent for their potential to improve outcomes in early-stage HCC [176]. This evolving landscape suggests a paradigm shift towards integrating ICIs into earlier treatment lines, aiming to optimize patient outcomes by targeting the disease at its most vulnerable stages.

### 6.6 Future Therapeutic Strategies and Directions

Beyond current systemic therapies, the future landscape of HCC treatment is rapidly evolving with novel immunotherapeutic approaches aiming to induce more robust and specific anti-tumor responses.

Tumor neoantigen strategies and immunopeptidomics identify patient-specific, immunogenic targets for personalized therapies, although the low tumor mutational burden in HCC remains a key challenge [143,177]. Vaccine platforms—including dendritic cell, mRNA, peptide (AFP, GPC3, MRP3), DNA plasmids with IL-12, and viral vectors, are being developed to prime or boost anti-tumor immunity, showing promise in preclinical and early clinical studies, particularly when combined with ICIs [131,178].

Furthermore, adoptive cell therapies represent a significant frontier, encompassing Chimeric Antigen Receptor T (CAR-T)-cell, T-cell Receptor engineered T (TCR-T)-cell, and Tumor-Infiltrating Lymphocyte (TIL), cytokine-induced killer (CIK) cells, natural killer (NK) cells, including CAR-NK, and others such as lymphokine-activated killer cells or gamma delta T cells.

CAR-T cells are engineered to recognize specific surface antigens, such as GPC3 [179]. In contrast, TCR-T cells can target intracellular antigens, including AFP and HBV-related [180]. Tumor-infiltrating lymphocyte (TIL) therapy involves expanding a patient’s own tumor-reactive lymphocytes *ex vivo* and then reinfusing them to enhance recurrence-free survival. Cytokine-induced killer (CIK) cells, which possess both T cell and NK cell characteristics, have demonstrated prolonged progression-free and overall survival in phase III trials following resection or locoregional therapy [181]. NK and CAR-NK cells leverage the liver’s high abundance of NK cells to mediate cytotoxicity independent of MHC interactions. Ongoing clinical trials are currently targeting GPC3 with these methods [182].

Although these advanced cellular therapies have shown efficacy in hematologic malignancies, their application in solid tumors such as HCC is challenged by the immunosuppressive tumor microenvironment [138]. Strategies to overcome these barriers include combining checkpoint blockade with cellular therapies, engineering T/NK cells for resistance to PD-1 exhaustion or TGF-β, lymphodepletion, or locoregional delivery [134,135].

Other emerging modalities include oncolytic viruses (e.g., engineered herpes simplex virus or vaccinia virus like Pexa-Vec), which selectively lyse tumor cells, and enhance immune responses, often synergizing with ICIs [28]. Bispecific T-cell engagers or antibodies, redirecting T cells or modulating dual pathways (tumor antigen–CD3, PD-1/CTLA-4), show early promise in preclinical HCC models [28,134]. Additionally, microbiome modulation and antibody-drug conjugates targeting HCC-specific antigens are also under exploration [183].

A critical unmet need remains the identification of robust, prospectively validated predictive biomarkers for patient selection and treatment decisions. Efforts focus on nomograms integrating clinical and molecular features, including GPC3, AFP, and activated β-catenin, Early declines in serum AFP correlate with improved response and survival after ICI therapy [184,185], while Wnt/β-catenin pathway alterations are linked to resistance [186]. Building effective biomarker-driven strategies is paramount for optimizing immunotherapy efficacy and improving patient selection and ultimately, improving patient survival. Many of these evolving strategies are currently in various phases of clinical trials, continuously shaping the treatment paradigm for HCC.

## 7 Challenges and Unmet Needs in Hepatocellular Carcinoma Treatment

Despite significant advancements in therapeutic strategies, HCC treatment continues to face formidable challenges that limit widespread efficacy and equitable patient access, underscoring the urgent need for further innovation.

In Particular, a critical gap persists in biomarker development for treatment selection, marked by the lack of robust, prospectively validated predictive biomarkers for patient selection. Building effective biomarker-driven strategies is paramount for optimizing immunotherapy efficacy, improving patient selection, and mitigating unnecessary toxicities, as the absence of reliable biomarkers currently hinders personalized treatment and contributes to suboptimal outcomes.

The HCC treatment landscape, despite recent advancements, features a limited number of effective systemic therapies, necessitating the identification of more novel therapeutic targets. Further studies are required to clarify the mechanisms underlying treatment resistance and to optimize standardized second-line options and combination therapies. Effective management of liver cancer demands multidisciplinary strategies that foster collaboration among diverse stakeholders, including patients, relatives, physicians, medical practitioners, regulatory bodies, and health authorities, to ensure optimal patient care.

Furthermore, health equity in access to advanced therapies remains compromised by global disparities in healthcare infrastructure, economic resources, and regulatory frameworks, which impede patient access to novel and expensive treatments. The high cost of innovative therapies further hinders widespread accessibility and risks exacerbating health inequities. Strategies to improve access to care and clinical trials in currently underrepresented regions are urgently needed.

## 8 Conclusion

The treatment landscape for HCC has undergone a profound transformation, moving from historically limited options to a sophisticated and evolving array of diagnostic and therapeutic strategies. Significant progress in systemic therapies for advanced disease, particularly the introduction of ICIs and novel combination regimens like atezolizumab-bevacizumab, has revolutionized patient outcomes. This evolution includes promising bispecific antibodies, such as PD-1/VEGF, PD-1/CTLA-4, and PD-1/LAG-3, and the anticipated shift towards triplet systemic therapy combining ICIs, anti-VEGF agents, and second-generation ICIs for advanced metastatic disease.

Beyond advanced stages, there is a growing interest in integrating ICIs into earlier disease settings, including neoadjuvant and adjuvant contexts, capitalizing on the potential for immune engagement when tumor burden is more manageable. Early studies in these perioperative settings have shown encouraging results, with major pathological responses observed, drawing parallels with successful applications in other cancers like melanoma and non-small cell lung cancer.

Despite these monumental advancements, critical challenges persist. A paramount unmet need is the development of robust, prospectively validated predictive biomarkers to optimize patient selection and guide treatment strategies. While biomarkers like GPC3, AFP, and activated β-catenin are under investigation, their reliable application is crucial for optimizing immunotherapy efficacy and minimizing unnecessary toxicities. Furthermore, global disparities in healthcare access and the high cost of innovative therapies present significant hurdles to equitable patient care. The future of HCC treatment will continue to be shaped by ongoing efforts to uncover molecular mechanisms, develop precision biomarker-driven strategies, and explore next-generation immunotherapies and targeted approaches to overcome resistance, ultimately contributing to improved patient longevity and enhanced health-related quality of life. Recent updates from the 2024 ASCO annual meeting highlight progress in ICIs, CAR-T cell therapies, oncolytic viruses, and locoregional combinations [187], while emerging triplet regimens, bispecific antibodies, adoptive T-cell therapies, and cancer vaccines offer exciting future possibilities [131]. Finally, as HCC affects diverse populations across the globe, ensuring equitable access to treatment is essential to reduce the global disease burden.

## Data Availability

Not applicable.
